# Surveillance Lessons from First-wave Pandemic (H1N1) 2009, Northern California, USA

**DOI:** 10.3201/eid1603.091285

**Published:** 2010-03

**Authors:** Roger Baxter

**Affiliations:** Kaiser Permanente Vaccine Study Center, Oakland, California, USA

**Keywords:** Influenza, H1N1, surveillance, California, pandemic, pandemic (H1N1) 2009, expedited, dispatch

## Abstract

After the appearance of pandemic (H1N1) 2009 in April 2009, influenza activity was monitored within the Kaiser Permanente Northern California division by using laboratory, pharmacy, telephone calls, and utilization (services patients received) data. A combination of testing and utilization data showed a pattern of disease activity, but this pattern may have been affected by public perception of the epidemic.

In April 2009, the novel swine-origin H1N1 influenza virus, now referred to as pandemic (H1N1) 2009 virus, was identified in the United States in California and in Mexico. During April, increasing numbers of cases were identified in Mexico, and sporadic cases were seen in the United States, mostly in returning travelers (*1*). Media coverage was high, and the public and medical communities were alert to the presence of the novel virus (*2*). Although the World Health Organization raised its influenza alert level to phase 6 (calling this a true pandemic) on June 11, by this time media attention in the United States had waned, and concern was for reemergence in the fall (*3*). However, virus activity did not diminish in northern California; rather, pandemic (H1N1) 2009 influenza remained active at high levels.

Kaiser Permanente (KP) is a medical care organization with 3.2 million members in its Northern California division (KPNC). Members receive essentially all medical care from KP providers and in KP facilities. An electronic medical record system records diagnoses from outpatient and emergency department visits and hospitalizations, as well as medications, immunizations, and ancillary services received by patients. A central laboratory in Berkeley performs all microbiologic and virologic testing. In addition, all telephone callers to the system are routed to central call centers, where information is gathered on whether the caller is asking influenza-related questions. This report details the recent experience of pandemic (H1N1) 2009 in KPNC and documents KP surveillance efforts.

## The Study

Influenza testing was performed by using a real-time PCR for influenza A and B and respiratory syncytial virus on nasopharyngeal swabs. Similar methods have been shown to be superior to other tests and sensitive and specific for detecting pandemic (H1N1) 2009 influenza (*4*). A weekly report went to primary care providers, advising on current viral activity, and gave guidelines for testing and treating. All specimens positive for influenza A were transported to the California State Department of Public Health laboratory for H1N1 confirmation testing early in the pandemic, but testing was later restricted to specimens from hospitalized patients only. Results of testing were provided weekly with counts from the previous week, Sunday through Saturday. Hospitalization rates were counted weekly by using text strings from admission diagnoses for pneumonia or influenza. If KPNC members had questions regarding influenza, when they called for advice or appointments they were triaged to the “flu queue,” where they could receive prerecorded messages or one-on-one advice with a nurse or physician. We plotted the percentage of all calls per week that were counted as influenza related. Weekly counts of medical office visits for influenza-like illness (ILI)—fever, influenza, or upper respiratory infection—were also plotted. The study was reviewed and approved by the Kaiser Permanente and our Institutional Review Boards.

**Figure F1:**
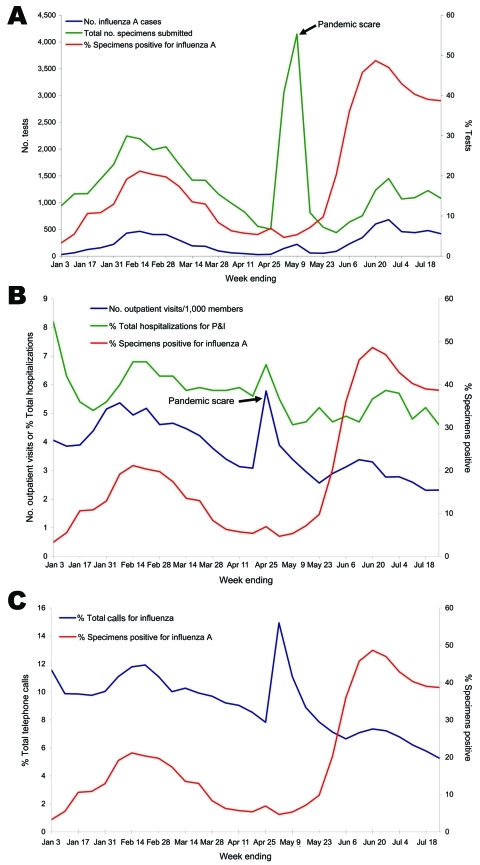
A) Influenza A testing in Kaiser Permanente Northern California division (KPNC), 2009. Shown are total numbers of specimens sent, number of specimens positive for influenza A, and percentage of specimens positive for influenza A. B) Outpatient visits for influenza-like illness (fever, influenza, or upper respiratory infection) per 1,000 members, percentage of all hospitalizations with a diagnosis of pneumonia or influenza (P&I), and percentage of specimens positive for influenza A, KPNC, 2009. C) Influenza-related telephone calls to KPNC, 2009, and percentage of specimens positive for influenza A.

The Figure, panel A, shows that the total number of respiratory tests rose drastically during late April, when media coverage was high. This increase was accompanied by an increase in the total number of respiratory specimens positive for influenza A. During this initial phase, utilization of resources was high, but there appeared to be little pandemic (H1N1) 2009 in the community because the percentage of positive specimens ranged from 5% to 7%. The Figure, [Fig F1]panel B, shows outpatient visits for ILI per 1,000 members and percentages of total hospitalizations for pneumonia or influenza during 2009, along with the percentage of respiratory specimens positive for influenza A. The first increase correlates with 2008–09 seasonal influenza, which peaked in February. Then in late April, at the same time as the increase in volume of influenza testing, there was an increase in outpatient visits for ILI and hospitalizations for pneumonia or influenza. In the Figure, panel C, the percentage of influenza-related telephone calls is plotted alongside the percentage of respiratory specimens that were positive for influenza A. Similarly to trends in medical appointments and hospitalizations, calls showed a marked increase during the pandemic scare period, then decreased and rose again more gradually with the first wave of the pandemic, along with the percentage of specimens positive for influenza A.

Media coverage rapidly subsided, and reports from the Centers for Disease Control and Prevention showed that the number of cases of pandemic (H1N1) 2009 was diminishing in Mexico and the United States (*1*). Testing and treating diminished and utilization of healthcare services returned to normal. However, pandemic (H1N1) 2009 continued to circulate widely, even after schools closed for summer vacation. By mid-May, the percentage of specimens positive for influenza A was 10% and then rapidly increased to 49% only 4 weeks later. State subtyping of hospitalized patients (inside and outside the KP) who were positive for influenza A showed that >95% of specimens tested from those patients were either not subtypeable or were positive for pandemic (H1N1) 2009 influenza virus (J. Louie, California Department of Public Health, pers. comm.). Hospitalizations for pneumonia and influenza, outpatient visits for ILI, and influenza-related phone calls all rose in concert with the percentage of positive specimens.

## Conclusions

During the recent outbreak of pandemic (H1N1) 2009 influenza in California, KPNC providers had access to quality, real-time information on the ongoing outbreak. This accessibility proved useful for guiding testing and treating algorithms and provided information during a time of great uncertainty and public fear.

Although the data were useful, it appears that during a time of intense media attention healthcare utilization may be susceptible to public perception and media coverage. During the pandemic scare period, although it appeared that influenza was circulating widely, test results and utilization data indicate that most activity was not related to either pandemic or seasonal influenza but that it may have been generated by demand created by false perceptions. It is interesting that even hospitalizations increased during this time because we generally perceive increased hospitalizations to be a marker of virulence and true activity. During the later phase of the pandemic, hospital and outpatient utilization rose in concert with the percentage of positive test results, reflecting virus activity. During this time, media coverage was relatively low, and this was reflected by lower numbers of telephone calls to the system.

The percentage of positive specimens appeared to be the best indicator of influenza activity because it was sensitive to rapid changes, but was a more specific indicator than specimens sent, number positive, outpatient or inpatient utilization, or telephone call-ins. The total number of patients tested is also informative because it can help define the relationship of testing to public perceptions. However, extremely high numbers can obscure a higher percent positive if persons seek medical care more from panic than for actual symptoms. The first-wave pandemic peak of positive samples was high compared with those from the seasonal influenza outbreak in February (49% vs. 22%); total numbers were lower. This difference may reflect patterns of testing by providers and reasons for patients to go to medical centers, but the high percentage of positive samples may reflect large numbers of cases in the community and the wide distribution of pandemic (H1N1) 2009 influenza.

The weekly report influenced provider testing with guidelines that changed as the season progressed. When the percentage positive was high at all facilities and the laboratory was overwhelmed with requests, providers were advised to decrease testing unless needed for a clinical workup or for any hospitalization. This request may have produced artifacts in the testing in that total numbers and the percentage positive may have varied based on the sensitivity and specificity of provider testing.

Surveillance for influenza, both seasonal and pandemic, by using electronic data is informative for medical organizations with a systematic approach to testing for influenza virus. Monitoring of medical utilization may be helpful in a pandemic, but fluctuations are susceptible to public impression and media coverage. An integrated approach to influenza surveillance, combining laboratory testing and utilization, would be optimal.
